# Role of Melatonin, Galanin, and RFamide Neuropeptides QRFP26 and QRFP43 in the Neuroendocrine Control of Pancreatic β-Cell Function

**DOI:** 10.3389/fendo.2017.00143

**Published:** 2017-07-03

**Authors:** Iacopo Gesmundo, Tania Villanova, Dana Banfi, Giacomo Gamba, Riccarda Granata

**Affiliations:** ^1^Division of Endocrinology, Diabetes and Metabolism, Department of Medical Sciences, University of Turin, Turin, Italy

**Keywords:** neurohormones, melatonin, galanin, QRF26, QRFP43, pancreatic β-cells

## Abstract

Glucose homeostasis is finely regulated by a number of hormones and peptides released mainly from the brain, gastrointestinal tract, and muscle, regulating pancreatic secretion through cellular receptors and their signal transduction cascades. The endocrine function of the pancreas is controlled by islets within the exocrine pancreatic tissue that release hormones like insulin, glucagon, somatostatin, pancreatic polypeptide, and ghrelin. Moreover, both exocrine and endocrine pancreatic functions are regulated by a variety of hormonal and neural mechanisms, such as ghrelin, glucagon-like peptide, glucose-dependent insulinotropic polypeptide, or the inhibitory peptide somatostatin. In this review, we describe the role of neurohormones that have been less characterized compared to others, on the regulation of insulin secretion. In particular, we will focus on melatonin, galanin, and RFamide neuropeptides QRFP26 and QRFP43, which display either insulinotropic or insulinostatic effects. In fact, in addition to other hormones, amino acids, cytokines, and a variety of proteins, brain-derived hormones are now considered as key regulators of glucose homeostasis, representing potential therapeutic targets for the treatment of diabetes and obesity.

## Introduction

Type 1 and type 2 diabetes (T2D) are characterized by a reduced insulin secretion from the pancreas, due to shortage of β-cells and decreased β-cell function. Because both types of diabetes eventually lead to β-cell loss, a major goal in research is to identify strategies to preserve β-cell mass and increase β-cell function (1, 2). Pancreatic exocrine and endocrine secretion is partly controlled by neuronal projections from the vagus nerve, as well as many hormones produced in peripheral tissues, including the gastrointestinal tract. These comprise the gastric peptide ghrelin, the intestinal peptides glucagon-like peptide 1 (GLP-1) and glucose-dependent insulinotropic polypeptide, somatostatin, produced by pancreatic δ-cells, or the adipose tissue-derived peptide leptin. Insulin release by β-cells is also influenced by non-hormonal signals, such as small proteins, amino acids, lipids, and cytokines. Moreover, recent studies have demonstrated that different neuropeptides are implicated in the regulation of glucose homeostasis and β-cell function, providing a physiological link between the brain and the endocrine pancreas (3, 4).

In the present review, we describe the role of neurohormones whose effects on insulin secretion and glucose homeostasis have been less well characterized compared to others. These include neuropeptides mainly displaying inhibitory functions on insulin secretion, such as the chronobiotic hormone melatonin, produced in the pineal gland, and galanin, released by the central and peripheral nervous systems and the gastrointestinal tract. Furthermore, we illustrate the effects of the hypothalamic RFamide peptides QRF26 and QRFP43, which, in addition to regulating feeding behavior, display both insulinostatic and insulinotropic actions and also promote pancreatic β-cell survival. Overall, because of their different ability to regulate β-cell function and glucose homeostasis, these hormones may be considered as potential therapeutic agents in diabetes and metabolic diseases.

### Melatonin

Melatonin is a hormone predominantly produced by the pineal gland of the mammalian brain. It is synthesized and secreted in a circadian manner at night and functions as chronobiotic agent, regulating the seasonal and circadian rhythms, such as the sleep–wake cycle. Therefore, it is a “Zeitgeber,” entraining circadian rhythm and indicating the time of day to various different organs and tissues in the body (5). In addition to the pineal gland, melatonin is produced by neuroendocrine cells in the retina and peripheral tissues, such as gastrointestinal tract, pancreas, and immune cells. In fact, because of its widespread production, melatonin acts in both endocrine and paracrine/autocrine manner. Furthermore, its effects have been shown in the cardiovascular and immune system, and on the regulation of metabolic functions (6–8).

At the cellular level, melatonin signals through two inhibitory G-protein (Gi)-coupled receptors, MT1 and MT2, whose binding results in inhibition of cAMP production. These receptors are widely distributed in the brain as well as in peripheral tissues, including the pancreas (9, 10). Furthermore, melatonin binding sites in cell nuclei of rat liver hepatocytes have been demonstrated (11) and identified as retinoid-related orphan receptor, mediating the genomic effects of the hormone (12, 13). Melatonin also interacts with cytosolic proteins, including calmodulin and calreticulin, implicated in the regulation of the cytoskeleton and the control of nuclear receptors (14, 15).

Interestingly, a variant of the human melatonin receptor 1 b gene (*MTRB1*) has been associated with high plasma glucose levels, reduction of insulin response to glucose, and increased risk of T2D (16–18). However, the role of melatonin on insulin secretion has not been clearly elucidated, as both inhibitory and stimulatory actions have been reported, probably because of the pleiotropism at the level of the receptor and second messengers (10, 19). Interestingly, most studies suggest that melatonin inhibits insulin secretion from pancreatic β-cells (20–22), while there are reports showing lack of effect (23). In fact, in INS-1 pancreatic β-cells, expressing MT1 receptors, acute treatment with melatonin inhibited GLP-1-induced insulin secretion. However, prolonged pretreatment with melatonin, enhanced insulin secretion in the presence of either the cAMP activator forskolin or GLP-1. Similar findings were observed in isolated rat islets (24). In another study, Peschke et al. demonstrated that melatonin inhibits cAMP and insulin secretion in INS-1 β-cells stimulated with forskolin, in a Gα_i_-dependent manner. Melatonin also inhibited insulin release in INS-1 cells treated with the inositol trisphosphate stimulator carbachol; however, in pertussis toxin (PTX)-incubated cells, the hormone increased carbachol-induced insulin release. These results suggested that in β-cells, MT1 receptor activates different signaling pathways displaying opposite effects on insulin secretion (25). Interestingly, downregulation of MT1 receptor expression in INS-1 β-cells reduced the insulinostatic effect of melatonin, indicating that, at least in rodent β-cells, the effects of the hormone are mainly mediated by this isoform of the receptor (26). Recently, rat islets and INS-1 cells were found to express MT2 (27), which is also involved in the inhibitory effect of the hormone on insulin secretion (27, 28). Of note, in isolated human pancreatic islets expressing both MT1 and MT2, melatonin promotes insulin secretion, in contrast with the effects in rodent β-cells and islets, possibly through an indirect action involving stimulation of glucagon secretion following its binding to MT1 receptors (29). In addition, melatonin has been shown to promote the secretion of glucagon in pancreatic αTC1.9 α-cells, expressing MT1 and MT2, treated with different concentrations of glucose (30). Furthermore, long-term administration of melatonin resulted in elevation of plasma glucagon concentrations in Wistar rats (WR), whereas in type 2 diabetic Goto-Kakizaki rats glucagon levels were decreased compared to untreated animals (30). Interestingly, mRNA expression for glucagon receptor, which was slightly reduced in the liver of untreated GK rats compared to WR, was upregulated by melatonin in GK rats and decreased in WR. Furthermore, MT1 and MT2 mRNA was elevated in the liver of MT1 or/and MT2 knockout (KO) mice compared to wild-type animals, suggesting that melatonin influences pancreatic glucagon secretion and displays metabolic effects in the liver.

With regard to melatonin and glucose homeostasis, it has been demonstrated that high levels of melatonin, due to blindness (31) or to exogenous administration of melatonin, result in an increase in blood glucose levels (32); moreover, glucose levels are reduced and insulin levels increased after pinealectomy (33, 34). However, most studies suggest that the pineal gland has an inhibitory effect on pancreatic β-cell function, as melatonin reduces insulin levels and glucose tolerance in animals and humans (35–38). Furthermore, elevation of insulin has been shown to inhibit the synthesis of melatonin from the pineal gland (39). Collectively, these findings suggest an antagonism between insulin and melatonin functions. This is further sustained by the fact that in man, insulin levels are elevated during the day and low at night, whereas the opposite occurs for melatonin (40); interestingly, diabetic patients show an abnormal circadian rhythm of melatonin (5). In addition, melatonin has been shown to promote the expression and release of GH and prolactin in female primates through MT1 (41), and the secretion of prolactin in humans (42–44), whereas ACTH secretion was found to be inhibited in the mouse pituitary corticotrope tumor cell line AtT20 (45). Hence, some of the actions of melatonin on glucose metabolism may be mediated by its effects on secretion of pituitary hormones.

A recent study has demonstrated that the risk variant rs10830963 of MTNR1B is an expression quantitative trait locus (eQTL), conferring increased expression of MTNR1B mRNA in human islets, which likely results in a reduction in insulin secretion and increased risk of T2D (22). Furthermore, melatonin was found to inhibit cAMP levels and insulin secretion in INS-1 832/13 β-cells, and these effects were further enhanced in β-cells overexpressing MTNR1B (22). Of note, melatonin is a prescription drug for improving sleep and for jet lag (8); therefore, it should be carefully administered in individuals with sleep disturbances, particularly in obese patients and carriers of the MNTR1B risk allele. However, administration of melatonin has been shown to improve sleep quality independently of rs10830963 genotype, despite the negative effect on insulin secretion (22). Moreover, the reduction of insulin release at night, mediated by the high levels of melatonin, when the metabolic demands are low because of reduced food intake, may be a protective physiological mechanism to prevent nocturnal hypoglycemia (22).

Interestingly, mice with a disruption of the receptor have been shown to secrete more insulin, despite no change in glucose levels, suggesting reduced insulin sensitivity but unchanged insulin tolerance (22). In addition, melatonin treatment in a human recall-by-genotype study was found to reduce insulin secretion in all subjects and to increase glucose levels; moreover, insulin reduction was even enhanced in individuals with the risk variant (22). Collectively, these findings suggest that increased melatonin signaling in islets impairs β-cell function, resulting in hyperglycemia and increased risk of T2D.

### Galanin

Galanin, a 29- to 30-amino acid neuropeptide initially discovered in porcine intestine (46), is expressed in the central and peripheral nervous systems and intestinal neuroendocrine system of many mammalian species (47–51). Galanin co-localizes and is coexpressed in neurons with a number of neurotransmitters and displays strong inhibitory effect on synaptic transmission (52–55). Because of its broad expression, galanin regulates many neuronal functions, such as memory and learning, neuropathic pain, neuroprotection, and neuroendocrine activity, representing a therapeutic potential for diseases such as Alzheimer’s disease, epilepsy, and diabetes (51, 56–58). Three distinct G-protein-coupled receptors GalR1, GalR2, and GalR3 are involved in the effects of the neuropeptide. GalR1 and GalR3 are coupled to the inhibitory G-protein Gi, whereas GalR2 associates with either Gi or Gq/11, thus displaying both inhibitory or stimulatory responses (51, 59).

Galanin-positive nerve fibers have been shown in the pancreas of different species, including rat, mouse (60, 61), and humans (62–64). Furthermore, a number of studies have indicated that galanin displays strong inhibitory effects on insulin secretion. In fact, galanin administration was found to reduce insulin levels in many species (65–67). In addition, a whole-genome profile study has demonstrated that the expression levels of a number of hippocampal genes, including galanin, and from the prefrontal cortex, such as GalR2, were dysregulated in type 2 diabetic rats, further suggesting the importance of the galanin system and the complexity of insulin signaling in modulating brain functions (68). Interestingly, infusion of galanin into animals through the pancreatic artery, at a concentration similar to that released from stimulated pancreatic nerve termini, resulted in inhibition of insulin secretion (69). However, conflicting results have been reported in humans, as galanin either suppresses insulin levels (70) or has no effect (71, 72). Moreover, galanin levels were inversely correlated with plasma insulin levels in postmenopausal women, whereas in controls there was a positive correlation (73).

Galanin and galanin analogs have been shown to reduce glucose-induced insulin secretion in isolated rat and pig islets (66, 74–76). The inhibitory action on insulin secretion in rat and mouse islets was found to involve a G_o2_ protein, through the regulation of both K_ATP_ and Ca^2+^ channels (60, 77). In line with these inhibitory effects, galanin infusion increased the levels of blood glucose in dogs but not in humans (69, 78). Furthermore, glucagon levels are upregulated by galanin, suggesting a role for glucagon in mediating the effects of galanin in glucose increase (49, 69).

Of note, transgenic mice overexpressing galanin showed visceral adiposity, increased body weight, increased serum cholesterol and triglycerides, hyperinsulinemia, and impaired glucose tolerance, indicating that elevated circulating galanin levels contribute to the development of metabolic syndrome (79). The obese phenotype was observed in the absence of increased food intake, suggesting defects in energy expenditure, since these mice had reduced oxygen consumption, as well as carbon dioxide and heat production (79). Surprisingly, mice with a loss-of-function mutation in the galanin gene [galanin KO mice] showed impaired inhibition of insulin secretion after activation of autonomic nerve, suggesting that galanin may act on sympathetic nerves to inhibit insulin secretion (80). Furthermore, insulin secretion was found reduced in galanin KO mice in response to glucose and arginine, compared to wild-type mice, and β-cells showed reduced sensitivity to glucose (80). Collectively, these findings suggest that in addition to regulating energy expenditure, galanin may be involved in the regulation of normal β-cell function. Conversely, galanin infusion has no effect on glucose tolerance in humans (71, 81, 82) and does not influence the postprandial rise of plasma glucose levels (70).

Reduced levels of pancreatic galanin were found in obese, hyperinsulinemic mice (83), and galanin-expressing cells were found to be strongly reduced in islets of diabetic rats (61). Interestingly, in rat and bovine pancreatic islets, galanin-like immunoreactivity co-localized with that of insulin, suggesting that galanin may influence insulin secretion in an autocrine/paracrine manner (61, 84). Furthermore, administration of a centrally active galanin analog with high affinity for GalR1 has been recently shown to reduce insulin secretion and promote hyperglycemia, providing a further understanding on the role of GalR1 *in vivo* (85).

However, a beneficial effect for galanin in animal models of diabetes has been also reported (86), therefore, additional studies are required to shed light on the role of galanin in human metabolic disorders and diabetes. Importantly, intranasally administered galanin-like peptide (GALP), whose aminoacid sequence 9–21 is identical to that of galanin 1–13, reduces body weight, food intake, water intake, and locomotor activity in leptin-deficient *ob/ob* mice and in diet-induced obese (DIO) mice (87). The decrease in body weight was found to be stronger in hyperglycemic compared to mormoglycemic mice, suggesting that intranasally administered GALP displays its best effect in obese mice with higher glucose levels. Interestingly, in DIO mice, the decrease in body weight after intranasal treatment with GALP was observed in spite of a reduction in locomotor activity, suggesting that GALP restrains energy intake and promotes energy expenditure (87). Other studies have demonstrated that intracerebroventricular GALP reduces food intake and stimulates energy expenditure; however, these effects did not persist over time, suggesting that the mice become insensitive to repeated treatment with GALP (88, 89). Conversely, repeated intranasal administration of GALP continued to decrease food intake and locomotor activity compared with repeated intracerebroventricular injection, suggesting that sensitivity to GALP is maintained and intranasal administration is the best way for GALP to exert its effects against obesity (87).

### RFamide Neuropeptide QRF26 and QRF43

The neuropeptide QRFP26 and its N-extended form QRFP43 are members of the RFamide peptide family, discovered in 2003 by three different groups (90–92). The gene encoding the QRFP26/QRFP43 precursor is widely distributed among vertebrates, including humans, mice, rats (90–92), and other species (93–95), indicating that these neuropeptides have been highly conserved during evolution (96).

QRFP26 and QRFP43 are the cognate ligands of the former orphan receptor GPR103, also called SP9155 or AQ27, and now renamed QRFPR (90, 97). QRFPR is a G-protein-coupled receptor, with a 52% amino acid identity with neuropeptide FF receptor 2 (NPFF2), another receptor for mammalian RFamide peptides. However, whereas QRFP26 also displays low moderate affinity for NPFF2, QRFP43 only binds to QRFPR, which, in turn, is not recognized by other mammalian RFamide peptides (98). Two isoforms have been described for QRFPR (QRFPR1 and QRFPR2) in rodents, sharing high homology with the unique form of human QRFPR, and QRFP26/QRFP43 bind with similar affinity to both forms of the receptor in rodents (99, 100).

The genes for QRFP26/QRFP43 precursor and QRFPR are mainly located in the hypothalamic nuclei, as well as in other brain areas involved in the control of feeding behavior (90, 101). Accordingly, intracerebroventricular (i.c.v.) injection of both QRFP26 and QRFP43 in mice has been shown to promote food intake and to increase body weight and fat mass (90, 97, 100, 102, 103). In addition to the central distribution, QRFP26/QRFP43 and QRFPR are expressed in peripheral organs, including adipose tissue and macrophages (104–106), eye, trachea, mammary gland, and testis, endocrine glands, including the pituitary, thyroid, and parathyroid glands, coronary artery, gastrointestinal tract, bladder, and prostate (91, 92, 100, 107). Thus, because of the broad distribution of QRFPR, QRFP26/QRFP43 have been shown to regulate a variety of physiological functions, including adipogenesis, lipolysis and inflammation (104–106), blood pressure (100), bone formation (108), and hypothalamo–pituitary–gonadal activity (109, 110).

Although initially not found in mouse and rat pancreas (91, 92), expression of QRFP26/QRFP43 and QRFPR mRNA and protein was later found in human endocrine pancreas and isolated pancreatic islets (107, 111), rat INS-1E β-cells (111), and mouse insulinoma MIN6 cells (107). Moreover, in human islets, QRFPR co-localized with insulin, suggesting autocrine/paracrine action of locally produced QRFP26/QRFP43 and direct binding of the peptides with its receptor in pancreatic β-cells (111).

Interestingly, QRFPR displays sequence similarity with NPY and galanin receptors (112), and like NPY and galanin, QRFP26/QRFP43 have been shown to regulate insulin secretion. In fact, QRFP26 was found to reduce glucose-, arginine-, and exendin-4-induced insulin secretion in rat perfused pancreas, showing no effect on glucagon secretion. Since the insulinostatic action of QRFP26 was inhibited by PTX upon treatment with exendin-4, it was suggested the involvement of a pertussis-sensitive Gα inhibitory (Gα_i_) protein negatively coupled to the adenylyl cyclase pathway (113). However, the authors of this study were unable to identify the receptor implicated in these effects, likely because previous reports failed to demonstrate QRFPR expression in the pancreas (91, 92).

In accordance with the findings of Egido et al. (113) QRFP26 was later found to inhibit glucose- and exendin-4-induced insulin secretion in INS-1E β-cells and human pancreatic islets, through mechanisms mediated by Gα_i_ and reduction of intracellular cAMP levels (111). Of note, knocking down QRFPR in these cells did not affect the insulinostatic action of QRFP26, suggesting the involvement of a different receptor. By contrast, QRFP43 potentiated insulin secretion in β-cells and human islets treated with both glucose or exendin-4, through engagement of a Gα stimulatory protein (Gα_s_) and elevation of cAMP levels (111). The insulinotropic effect of QRFP43 was suppressed when QRFPR was knocked down in INS-1E β-cells using small interfering RNA, whereas the insulinostatic effect of QRFP26a was maintained. Furthermore, QRFP43, but not QRFP26 increased glucose uptake by β-cells. At variance with the opposed effects observed on β-cell function, both peptides reduced apoptosis and cell death induced by serum starvation, inflammatory cytokines and glucolipotoxicity in β-cells and human islets, to an extent comparable to that induced by exendin-4. QRFP43-induced protection involved activation of the survival and proliferative pathways phosphatidylinositol 3-kinase/Akt and extracellular signal-related kinase 1/2 (ERK1/2), whereas only ERK1/2 was required for the survival function of QRFP26 (111). At present it is unclear why both QRFP26 and QRFP43 promote survival of β-cells, while having opposed effects on insulin secretion. The possible explanation would be that, in addition to QRFPR, these peptides bind to one or more yet unknown alternative receptors involved in their survival action.

The role of QRFP26 was recently investigated on the regulation of glucose homeostasis (107). It was demonstrated a positive association between the levels of plasma QRFP26 and plasma insulin in patients with diabetes; furthermore, QRFP26 increased in response to an oral glucose tolerance test. In mice, QRFP26 attenuated glucose-induced hyperglycemia, increased insulin sensitivity and plasma insulin concentrations but did not alter basal glycemia, suggesting antihyperglycemic action. In addition, QRFP26 promoted insulin secretion in MIN6 insulinoma cells, in a QRFPR-dependent manner, as inhibition of QRFPR expression using specific siRNA blocked the insulinotropic effect of the peptide. Accordingly, MIN6 showed expression for QRFPR but not for NPFF2, the other RFamide receptor that can be recognized by QRFP26. Conversely, in INS-1E β-cells the insulinostatic action of QRFP26 was independent QRFPR binding, suggesting that other receptor(s), such as NPFF2 would be involved. However, to date, the presence of NPFF2 in INS-1 β-cells or human pancreatic islets remains to be determined. Thus, the different effect of QRFP26 on insulin secretion in different β-cell types may be attributed to the different expression pattern of the receptor(s). Interestingly, in *both in vivo* and *in vitro* experiments, high concentrations of glucose induced a massive secretion of QRFP26 by the small intestine (107). Overall, at variance with the results of Granata et al. these findings indicated that QRFP26 acts as an incretin hormone to regulate glucose homeostasis.

Overall, the results from different reports indicate that QRFP26/QRFP43 regulate glucose homeostasis and β-cell function; however, further understanding is required to disentangle the discrepancies observed in the various experimental models and for elucidating the role of the receptor(s) involved in these effects. Of note, these neuropeptides increase the survival of β-cells and human pancreatic islet cells, suggesting potential therapeutic implications in diabetes.

## Conclusion

**Figure 1:**
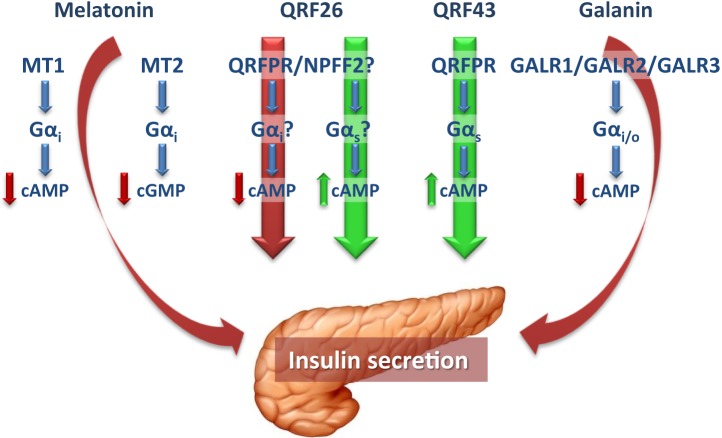
In pancreatic β-cells, the pineal hormone melatonin reduces insulin secretion through binding to the receptor isoforms MT1 and MT2, which, by coupling to Gα_i_ proteins, inhibit cAMP and cGMP, respectively. The hypothalamic peptide QRFP26 has been shown to both inhibit and promote insulin secretion in different β-cell models, through receptors that in part remain to be defined. The stimulatory and inhibitory actions of QRFP26 are likely mediated by activation of Gα_s_ and Gα_i_ proteins, respectively. On the other hand, QRFP43 acts as an insulinotropic neuropeptide by binding to a Gα_s_-coupled QRFPR, to increase cAMP levels. Galanin, widely distributed in both central and peripheral nervous systems, displays inhibitory effects in the endocrine pancreas and, like melatonin, reduces insulin secretion in β-cells by binding to Gα_i_-coupled receptors (GALR1-3). Red and green arrows indicate the inhibitory and stimulatory effects on insulin secretion, respectively. cAMP, cyclic adenosine monophosphate; cGMP, cyclic guanosine monophosphate; GALR, galanin receptor; Gα_i_, inhibitory guanine triphosphate-binding protein α-subunit; Gα_s_, stimulatory guanine triphosphate-binding protein α-subunit; MT1 and MT2, melatonin receptor 1 and 2; NPFF2, neuropeptide FF receptor 2; QRFPR, QRFP receptor.

Many important questions on the regulation of β-cell function remain unanswered, as a variety of players, and even more to be discovered, are implicated in this complex process. In addition to their central actions, it is becoming increasingly clear that, together with peripheral hormones, neuropeptides are also key regulators of glucose homeostasis and insulin secretion, displaying both direct and indirect actions in the endocrine pancreas (Figure 1). Thus, it is important to further understand their specific role and mechanisms, in order to increase the wide range of potential therapeutic targets for the treatment of diabetes and metabolic diseases.

## Author Contributions

IG, TV, DB, and GG contributed to the writing of the different topics and edited the manuscript; RG wrote the paper and supervised the work of the co-authors.

## Conflict of Interest Statement

The authors declare that the research was conducted in the absence of any commercial or financial relationships that could be construed as a potential conflict of interest.
